# A single dose of i.v. iron induces cardiac ferroptosis in murine cardiometabolic heart failure

**DOI:** 10.1172/jci.insight.195410

**Published:** 2026-02-26

**Authors:** Caitlin M. Pavelec, Leigh A. Bradley, Priyanka Rawat, Luke S. Dunaway, Maya Bolger-Chen, Bethany A. Gholson, Jonathan R. Lindner, Brant E. Isakson, Norbert Leitinger, Matthew J. Wolf

**Affiliations:** 1 Department of Medicine,; 2 Robert M. Berne Cardiovascular Research Center,; 3 Department of Pharmacology, and; 4 Department of Biophysics and Molecular Biology University of Virginia School of Medicine, Charlottesville, Virginia, USA.

**Keywords:** Cardiology, Cell biology, Heart failure, Molecular pathology

Heart failure with preserved ejection fraction (HFpEF) is a clinical syndrome causing symptoms including decreased exercise tolerance and dyspnea. Characterization of clinical phenogroups within patients with HFpEF using the standards from Treatment of Preserved Cardiac Function Heart Failure with an Aldosterone Antagonist Trial (TOPCAT) identified 1 of 3 that demonstrates cardiometabolic and inflammatory phenotypes (cardiometabolic HFpEF) (1). This patient population accounts for the largest healthcare burden and has the highest mortality of all groups.

In contemporary cohorts, iron deficiency is highly prevalent in HFpEF (approximately 50%–60%) and associated with worse symptoms, reduced exercise capacity, and higher mortality (2). In heart failure with reduced ejection fraction (HFrEF), iron infusion has been shown to improve patient outcomes with repeated administration. However, whether iron infusion in HFpEF is as beneficial as in HFrEF is less understood. Few trials have investigated this, and while the FAIR-HFpEF showed improved 6-minute walking distance, it was terminated early due to low recruitment limiting its ability to determine cardiac outcomes (3).

While the primary goal of iron infusion is to enhance HF outcomes, there is the potential for acute myocardial injury following iron infusion. Iron infusion could trigger bouts of ferroptosis, a regulated cell death characterized by iron-dependent oxidative damage and plasma membrane lipid peroxidation in a variety of lipid species (4). Therefore, we investigated if a single dose of i.v. iron could induce ferroptosis in the myocardium in the context of cardiometabolic HF.

C57BL/6 male mice were fed a high-fat high, high-sucrose (HFHS) diet or chow control, previously shown to decrease myocardial perfusion reserve, decrease longitudinal and diastolic strain rate, increase body weight, and impair glucose sensitivity phenocopying aspects of HFpEF (5). Mice fed HFHS or control chow received a single dose of i.v. iron dextran, equivalent to that given to patients. Forty-eight hours after i.v. iron, the hearts were analyzed (Figure 1A and Supplemental Methods). Hearts were perfused and divided for histology and lipidomics, and a subset of hearts was allocated for RNA-seq. RNA-seq revealed that, while HFHS feeding alone created a distinct grouping from mice fed chow, there is also a distinct grouping between mice on chow treated with i.v. iron and those on HFHS treated with i.v. iron (Supplemental Figure 1A; supplemental material available online with this article; https://doi.org/10.1172/jci.insight.195410DS1). Compared with vehicle, iron HFHS mice had increased expression of *Nppb*, a biomarker of HF exacerbation indicating increased cardiac damage (Supplemental Figure 1B). We also evaluated cardiovascular function in mice fed a HFHS diet that received iron and found a decrease in running time (Supplemental Figure 1B). HFHS-fed mice given i.v. iron also had a significant increase in heart mass and a change in E/A ratio as measured by echocardiography (Supplemental Figure 1B). This suggests that i.v. iron administration alters cardiovascular parameters in HFHS-fed mice. Hierarchal clustering of gene expression identified pathways, including lipid metabolism and calcium regulation, that were downregulated in HFHS mice after iron compared with controls. Conversely, positive regulators of oxidative damage and phagocytosis were upregulated, demonstrating a unique regulation of damage only in the case of HFHS-fed mice after i.v. iron but not their chow counterparts (Supplemental Figure 1C). Compared with iron mice on chow, the hearts of mice fed HFHS that received iron had increased the expression of genes associated with ferroptosis (Figure 1, B and C). More specifically, genes considered proferroptotic were upregulated in HFHS-fed mice after iron (Figure 1C).

We took 2 approaches to investigate ferroptosis in the hearts of mice fed HFHS that received iron. First, we examined the C_11_-BODIPY staining in cardiomyocytes of mice fed HFHS or control chow and given a single dose of IV iron. C_11_-BODIPY resides in lipophilic membrane structures where reactive enzymatic and nonenzymatic pro-oxidants can oxidize it (4) (Supplemental Methods). This is an indirect measure of the lipid ROS flux and has been used to detect ferroptosis. Cardiomyocytes of HFHS mice treated with iron had increased oxC11-BODIPY staining compared with chow controls (Figure 1, D and E). Second, we measured oxidized phospholipids (oxPLs) by liquid chromatography–tandem mass spectrometry (LC-MS/MS) in heart tissue (6). oxPLs across a panel of species compared with standard curves were increased in the hearts of mice fed a HFHS diet that received i.v. iron compared with HFHS-fed mice, consistent with ferroptosis (Figure 1F and Supplemental Figure 1D). This indicates both a deleterious cellular process and a pathological effect on cardiac function from a single dose of i.v. iron.

We identified that a single dose of i.v. iron in mice acutely induced changes consistent with myocardial ferroptosis. This discovery highlights the urgency of determining if repeated doses of i.v. iron induce repetitive bouts of ferroptosis and if this adversely affects cardiac function. Further studies should evaluate if the coadministration of ferroptosis inhibitors with i.v. iron mitigates potential harm while providing the benefits of iron supplementation in cardiometabolic HFpEF.

## Conflict of interest

The authors have declared that no conflict of interest exists.

## Funding support

This work is the result of NIH funding, in whole or in part, and is subject to the NIH Public Access Policy. Through acceptance of this federal funding, the NIH has been given a right to make the work publicly available in PubMed Central.

- NIH R01 HL158718 to MJW
- NIH T32 HL007284 to CMP

## Supplementary Material

Supplemental data

Supporting data values

## Figures and Tables

**Figure 1:**
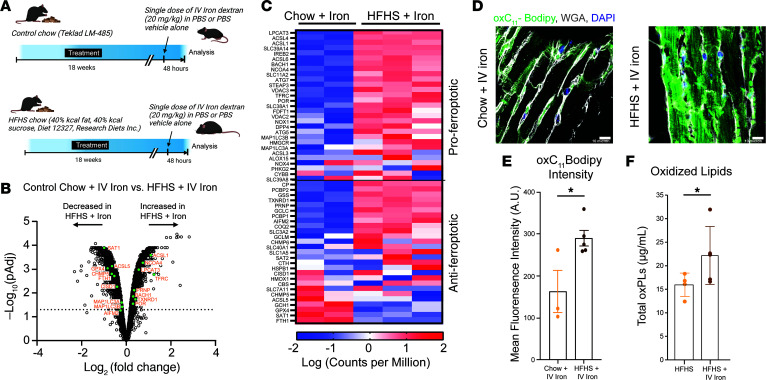
I.V. Iron induces cardiac ferroptosis in murine cardiometabolic heart failure. (**A**) Experimental protocol for mice fed control chow or HFHS diet and given a dose of IV iron. (**B**) Volcano plot of genes expressed in hearts of mice fed HFHS or chow and give iron (*n* = 2–3 samples per group). Solid line indicates a –log_10_(*P*_adj_) values of 1.3. Genes highlighted in green are significantly changed and included in the ferroptosis pathway (WikiPathways). (**C**) Heatmap of the expression of ferroptosis genes (WikiPathways) from hearts of mice fed control chow or HFHS that were given i.v. iron. Scale is –2 to +2, normalized for counts per million. (**D** and **E**) Confocal microscopy and quantification of oxC11-BODIPY staining of hearts of mice fed control chow or HFHS diet given IV. *n* = 3 control group and *n* = 5 HFHS group. Scale bars: 10 μm. *P* value by 2-tailed Student *t* test. (**F**) Quantification of total oxidized phospholipids by LC-MS/MS comparing hearts of HFHS diet that were given vehicle (*n* = 4) or i.v. iron (*n* = 5). *P* value by Student’s *t* test.

## Version 1 02/26/2026

In-Press Preview

## Version 2 04/22/2026

Electronic publication
