# Supplementary material for: A single dose of i.v. iron induces cardiac ferroptosis in murine cardiometabolic heart failure
Source: JCI Insight. 2026 Feb 26;11(8):e195410. doi: 10.1172/jci.insight.195410 (PMC13135405; doi:10.1172/jci.insight.195410)
Supplement: Supplemental data [file jciinsight-11-195410-s002.pdf]

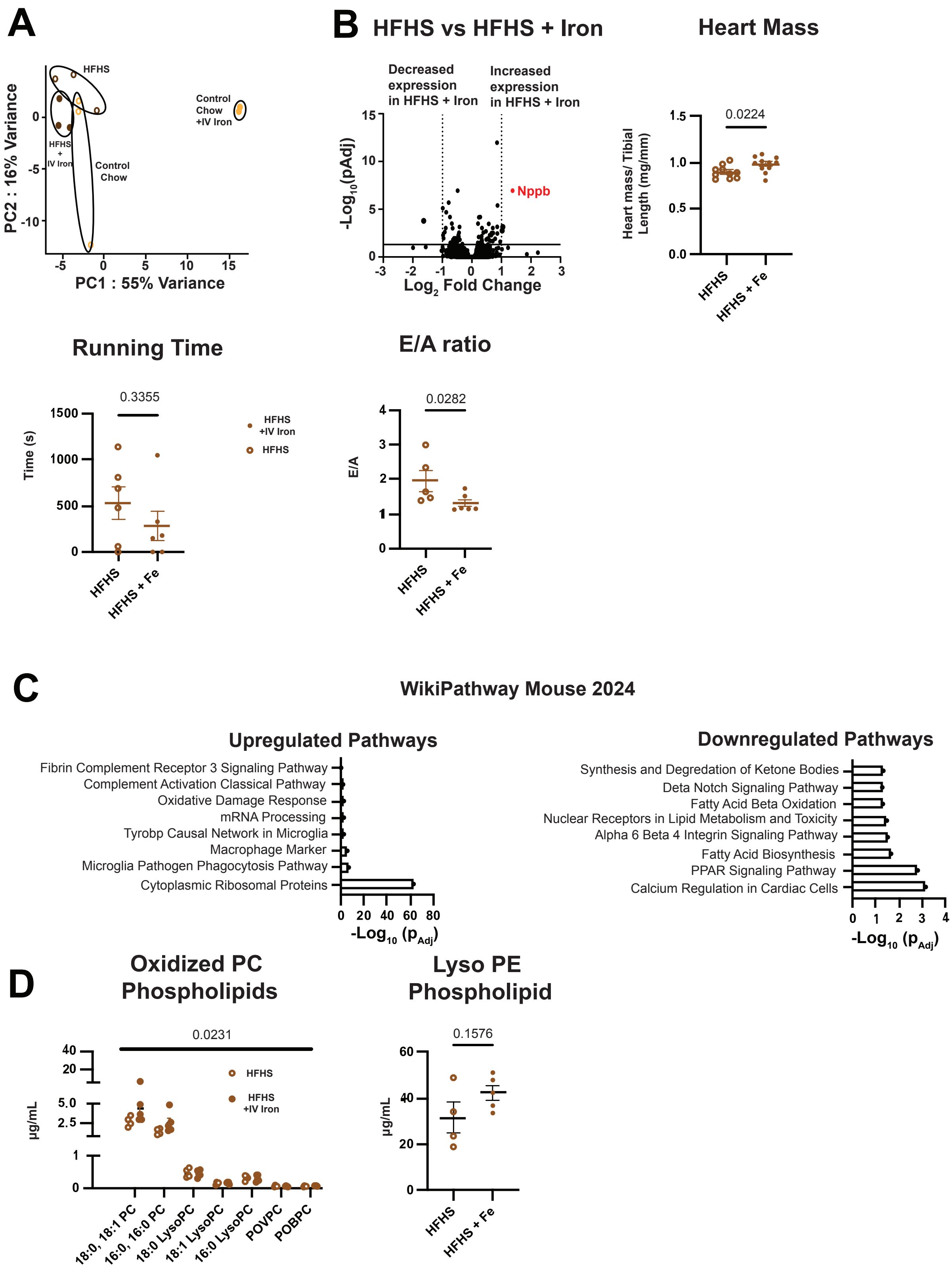
A
PC2 : 16% Variance
PC1 : 55% Variance
HFHS
HFHS + IV Iron
Control Chow
Control Chow +IV Iron
B
HFHS vs HFHS + Iron
Decreased expression in HFHS + Iron
Increased expression in HFHS + Iron
-Log10(pAdj)
Log2 Fold Change
Nppb
Heart Mass
Heart mass/ Tibial Length (mg/mm)
0.0224
HFHS
HFHS + Fe
Running Time
Time (s)
0.3355
HFHS +IV Iron
HFHS
E/A ratio
E/A
0.0282
C
WikiPathway Mouse 2024
Upregulated Pathways
Fibrin Complement Receptor 3 Signaling Pathway
Complement Activation Classical Pathway
Oxidative Damage Response
mRNA Processing
Tyrobp Causal Network in Microglia
Macrophage Marker
Microglia Pathogen Phagocytosis Pathway
Cytoplasmic Ribosomal Proteins
-Log10 (pAdj)
Downregulated Pathways
Synthesis and Degredation of Ketone Bodies
Deta Notch Signaling Pathway
Fatty Acid Beta Oxidation
Nuclear Receptors in Lipid Metabolism and Toxicity
Alpha 6 Beta 4 Integrin Signaling Pathway
Fatty Acid Biosynthesis
PPAR Signaling Pathway
Calcium Regulation in Cardiac Cells
-Log10 (pAdj)
D
Oxidized PC Phospholipids
µg/mL
0.0231
HFHS
HFHS +IV Iron
18:0, 18:1 PC
16:0, 16:0 PC
18:0 LysoPC
18:1 LysoPC
16:0 LysoPC
POVPC
POBPC
Lyso PE Phospholipid
µg/mL
0.1576
HFHS
HFHS + Fe

Supplemental Figure: **Figure Legend.** **(A)** PCA plot of the RNA expression of genes from hearts of the four groups. **(B)** Volcano plot of genes expressed in hearts of mice fed HFHS diets and given IV iron or placebo. (N = three samples per group). Solid line indicates a -log10(pAdj) values of 1.3. Dashed lines indicate a Log2 fold change of -1 and 1. Heart mass normalized to tibial length was measured from mice fed HFHS diet (N=10) compared with HFHS fed mice administered iron (N=11). Treadmill maximal running time was measured in mice fed HFHS diet (N= 6) versus HFHS fed mice administered iron (N=6). E/A measured by echocardiography was determined from mice fed HFHS diet (N=5) versus mice fed HFHS diet and administered IV iron (N=6). P-value was determined by student's t-test for all bar-whisker plots provided. **(C)** Pathway analysis (WikiPathways) of differentially expressed genes from mice fed HFHS diet compared to chow controls with IV iron administration. **(D)** Quantification of oxidized phospholipid species by LC-MS/MS comparing hearts of HFHS diet that were given vehicle alone (N = 4) or IV iron (N=5). p-value of 0.01 was determined by mixed effects analysis (2-way ANOVA). P-value was determined by student's t-test for Lyso-PE.

Methods

Sex as a biological variable: This study was performed using only male mice as indicated in the manuscript. The model of high fat high sucrose feeding used has only been previously published using male mice and therefore has not been established to induce cardiometabolic heart failure in both biological sexes.

Animals: C57Bl6/J male mice were procured from the Jackson laboratory (strain - 000664). Mice were maintained on a 12-hour light/dark cycle and unless otherwise described provided ad libitum access to standard rodent chow (Teklad 7912) or high fat, high sucrose diet (40% fat, 40% sucrose) (ResearchDiets D12327). Mice were randomized to diet or chow beginning at 12 weeks of age and maintained on diet for 18 weeks. After 18 weeks of feeding mice were subjected to

intravenous (IV) tail vein injection of PBS (vehicle) or iron dextran (20 mg/kg) (Sigma D8517) over a period of 2 minutes of administration. Mice were harvested 48 hours after injection and tissues analyzed by RNA-Sequencing or histopathology.

Treadmill Runing: Exercise capacity was assessed by a blinded investigator similar to previously published methods (PMID: 36302779). Mice were acclimatized to the treadmill for three consecutive days running at 13 m/min (0.5 mph) for 2 min. On the fourth day, mice were run at 13 m/min on a 5% incline until exhaustion. Three ping pong balls were placed at the back of each treadmill lane to motivate the mice. Exhaustion was defined as repeated failure to stay in front of the pings pong balls after three consecutive assists.

C_{11} – Bodipy Staining: Cardiac sections from mice fed HFHS diet or control chow and given IV iron were perfused with PBS and fixed in NBF for four hours before being subsequently paraffin embedded and sectioned at 7-10 µm. Sections were subjected to deparaffinization with xylene and antigen retrieval was performed (Vector Laboratories H-330). Sections were stained with C_{11}-BODIPY at 1:200 at room temperature for 1 hour per manufacture instructions. Oxidation of the polyunsaturated butadienyl portion of C_{11}-BODIPY (oxC_{11}-BODIPY) shifts the fluorescence emission peak from ~590 nm to ~510 nm. Sections were imaged on an Olympus Fluoview 1000 and are representative images of composite z-stacks. Analysis (thresholding and fluorescence intensity quantification) were performed in ImageJ.

RNA-Sequencing: Hearts of mice were removed and whole-body perfusion with 10mL of PBS, weighed, and snap frozen at -80C prior to analysis. Total RNA, library preparation including quality metrics, sequencing, and clustering was performed by Azenta/GeneWiz (South Plainfield, NJ). One hundred fifty-base paired-end sequencing was performed using an Illumina NovaSeq platform according to manufacture instructions. Fastq files were processed using in-house perl scripts from Genewiz to obtain clean reads. Clean reads were aligned to the mouse reference genome. Differential expression analysis was performed using DESeq2. Differentially expressed genes (DEG) were assigned by criteria of P-value adjusted < 0.05 (-Log(p-value) > 1.3).

Statistics: Statistical analyses were performed in Prism 10 (GraphPad). Statistical tests were performed with two-tailed analysis. Comparisons between two groups was conducted by Student's t-test, while comparisons between more than two groups were made by one-way or two-way analysis of analysis of variance where appropriate. Sidak's multiple comparisons test was performed as post-hoc analysis where appropriate. A p-value of less than 0.05 was considered statistically significant. Data are represented as mean plus minus the standard error of the mean. Image analysis was performed on 3-5 averaged images per mouse across a minimum of three cardiac sections. Data points shown for imaging are representative of the average across a mouse, with each data point representative of individual mice.

Study approval: All animals used for these studies were approved by the University of Virginia animal care and use committee (ACUC) under protocol number 4080.

Data Availability: Sequencing data reported in this manuscript are deposited in the NCBI Gene expression Omnibus (Accession Number: GSE313685). All data needed to evaluate the conclusions of this manuscript are present in the manuscript, present in the supplementary figure, or available upon request to the corresponding author. Individual data values are provided in the supporting data values file associated with this manuscript. Graphics were prepared using BioRender. This work is the result of NIH funding, in whole or in part, and is subject to the NIH Public Access Policy.

Conflict of Interest: No author has any conflict of interest regarding ownership, income, research support, or intellectual property.
